# Timing of High-Dose Rate Brachytherapy With External Beam Radiotherapy in Intermediate and High-Risk Localized Prostate CAncer (THEPCA) Patients and Its Effects on Toxicity and Quality of Life: Protocol of a Randomized Feasibility Trial

**DOI:** 10.2196/resprot.4462

**Published:** 2015-04-29

**Authors:** Sreekanth Palvai, Michael Harrison, Sharon Shibu Thomas, Karen Hayden, James Green, Oliver Anderson, Lavinia Romero, Richard Lodge, Patricia Burns, Imtiaz Ahmed

**Affiliations:** ^1^Southend University Hospital National Health Service Foundation TrustNational Health ServiceEssexUnited Kingdom; ^2^Anglia Ruskin - Clinical Trials UnitPostgraduate Medical InstituteAnglia Ruskin UniversityChelmsfordUnited Kingdom

**Keywords:** prostate cancer, radiotherapy, brachytherapy, external beam radiotherapy, EBRT, randomized controlled trial, RCT, Southend Hospital

## Abstract

**Background:**

Prostate cancer is the most common cancer in males in the UK and affects around 105 men for every 100,000. The role of radiotherapy in the management of prostate cancer significantly changed over the last few decades with developments in brachytherapy, external beam radiotherapy (EBRT), intensity-modulated radiotherapy (IMRT), and image-guided radiotherapy (IGRT). One of the challenging factors of radiotherapy treatment of localized prostate cancer is the development of acute and late genitourinary and gastrointestinal toxicities. The recent European guidelines suggest that there is no consensus regarding the timing of high-dose rate (HDR) brachytherapy and EBRT. The schedules vary in different institutions where an HDR boost can be given either before or after EBRT. Few centers deliver HDR in between the fractions of EBRT.

**Objective:**

Assessment of acute genitourinary and gastrointestinal toxicities at various time points to better understand if the order in which treatment modality is delivered (ie, HDR brachytherapy or EBRT first) has an effect on the toxicity profile.

**Methods:**

Timing of HDR brachytherapy with EBRT in Prostate CAncer (THEPCA) is a single-center, open, randomized controlled feasibility trial in patients with intermediate and high-risk localized prostate cancer. A group of 50 patients aged 18 years old and over with histological diagnosis of prostate cancer (stages T1b-T3BNOMO), will be randomized to one of two treatment arms (ratio 1:1), following explanation of the study and informed consent. Patients in both arms of the study will be treated with HDR brachytherapy and EBRT, however, the order in which they receive the treatments will vary. In Arm A, patients will receive HDR brachytherapy before EBRT. In Arm B (control arm), patients will receive EBRT before HDR brachytherapy. Study outcomes will look at prospective assessment of genitourinary and gastrointestinal toxicities. The primary endpoint will be grade 3 genitourinary toxicity and the secondary endpoints will be all other grades of genitourinary toxicities (grades 1 and 2), gastrointestinal toxicities (grades 1 to 4), prostate-specific antigen (PSA) recurrence-free survival, overall survival, and quality of life.

**Results:**

Results from this feasibility trial will be available in mid-2016.

**Conclusions:**

If the results from this feasibility trial show evidence that the sequence of treatment modality does affect the patients’ toxicity profiles, then funding would be sought to conduct a large, multicenter, randomized controlled trial.

**Trial Registration:**

International Standard Randomized Controlled Trial Number (ISRCTN): 15835424; http://www.isrctn.com/ISRCTN15835424 (Archived by WebCite at http://www.webcitation.org/6Xz7jfg1u).

## Introduction

### Disease Background

Prostate cancer is the most common cancer in males in the UK, affecting around 105 men for every 100,000 [1]. The role of radiotherapy (RT) in the management of prostate cancer significantly changed over the last few decades with developments in brachytherapy (BT), external beam radiotherapy (EBRT), intensity-modulated radiotherapy (IMRT), and image-guided radiotherapy (IGRT). One of the challenging factors of radiotherapy treatment of localized prostate cancer is the development of acute and late genitourinary and gastrointestinal toxicities. There are several studies and case series published in the literature assessing the toxicities developed during EBRT and brachytherapy treatment for prostate cancer.

### Background for Study

EBRT and brachytherapy emerged as the mainstays of localized prostate cancer treatment in recent years. Brachytherapy can be delivered either in low-dose rate (LDR) or in high-dose rate (HDR). The low-risk, localized prostate cancers can be treated with low-dose brachytherapy or by prostatectomy, whereas the intermediate and high-risk localized prostate cancers are usually treated with EBRT alone or in combination with HDR brachytherapy (HDR-BT). HDR monotherapy in this patient group is not routinely practiced unless as part of a study. Radiation dose escalation has been proven to be effective in biochemical response and clinical outcomes in prostate cancer. However, increased toxicity limits the total dose of radiation that can be safely administered [2-4]. Combining external beam radiotherapy with a brachytherapy boost has been effective in tumor control, allowing for significant dose escalation without any change in acute and late toxicities in comparison to external beam radiotherapy alone [5].

The relative sensitivity of radiotherapy depends on the alpha/beta ratio. This ratio expresses the sensitivity to radiation fraction size and estimates the impact of the given radiation schedule on tumor control and toxicity. There is increasing evidence to support the alpha/beta ratio for prostate cancer to be as low as 1.5 Gy. The evidence indicates that a hypofractionated radiation schedule—larger dose per fraction with smaller number of fractions—would offer optimal tumor control [6-9]. As a result, the practice of combining EBRT with HDR brachytherapy is gaining momentum in clinical practice. However, the current practice across the globe differs in both radiation doses and in the timing of each modality delivered.

The recent European guidelines suggest that there is no consensus regarding the timing of HDR brachytherapy and EBRT. The schedules vary in different institutions where an HDR boost can be given either before or after EBRT. Few centers deliver HDR in between the fractions of EBRT [10].

The EBRT doses range from 37.5 Gy in 13 fractions (2.88 Gy per fraction) to 45 Gy in 25 fractions (1.8 Gy per fraction) when given with HDR. The total HDR brachytherapy dose can be delivered in fractions, however a single dose of 15 Gy is gaining acceptance across the world due to its logistical advantage [10]. The time gap between the two radiotherapy modes of delivery is generally within 21 days.

The toxicity profile of radiation therapy is dependent on the type of modality used to deliver the treatment, and whether the treatment is delivered as a combined modality or standalone treatment [11]. A randomized phase III trial where EBRT was delivered before HDR showed that the 5- and 7-year incidence for patients with any severe urinary symptom was 26% and 31%, respectively, for those treated with EBRT and HDR-BT delivered sequentially. For patients given EBRT alone, the 5- and 7-year incidence was 26% and 30%, respectively (log rank *P*=.5). The incidence of severe bowel events for the EBRT/HDR combination group was considerably lower—7% and 6% at 5 and 7 years, respectively (log rank *P*=.8) [5]. On the other hand, a single-arm phase II study was performed to determine the toxicity profile of EBRT delivering a dose of 37.5 Gy in 15 fractions that was given after a single-fraction HDR boost of 15 Gy. In this study, Morton et al found acute grade 2 and grade 3 genitourinary toxicity in 62% and 1.6% of patients, respectively, and acute grade 2 gastrointestinal toxicity in 6.5% of patients, with no grade 3 gastrointestinal toxicity [12].

Both acute and late toxicity assessments in prostate cancer patients are assessed by various tools. For example, the European Organisation for Research and Treatment of Cancer Quality of Life Questionnaire for Cancer Patients (EORTC QLQ-C30), the European Organisation for Research and Treatment of Cancer Quality of Life Questionnaire for Prostate Cancer Patients (QLQ PR25), the National Cancer Institute Common Terminology Criteria for Adverse Events (CTCAE) grading system, the Functional Assessment of Cancer Therapy-Prostate (FACT-P), version 4, the International Prostate Symptom Score (IPSS), the International Index of Erectile Function Scale (IIEFS), and the Expanded Prostate Cancer Index Composite (EPIC) questionnaire may be used for toxicity assessments. Furthermore, the same data collection tools are used to measure health-related quality of life.

### Rationale and Risks/Benefits

There is no consensus about the timing of HDR brachytherapy when treating prostate cancer along with EBRT. The advantages of using HDR brachytherapy before EBRT are that patients could potentially be identified who are not suitable for brachytherapy early in the treatment process. As the patients would be radiotherapy naïve, there would be less chance of soft tissue injury during the brachytherapy process if BT is given first. However, having brachytherapy first can be logistically difficult in a busy radiotherapy unit in terms of planning and arranging delivery of EBRT within 2 to 3 weeks of BT. Moreover, if the patients develop acute urinary complications during brachytherapy they would need to continue with EBRT with a urinary catheter, which could potentially prolong the duration of the catheter in situ and cause significant patient discomfort.

On the other hand, delivering EBRT first is logistically easier to arrange and could theoretically make the hypofractionated radiation dose of brachytherapy more effective, as tumor cells could become more radiosensitive due to molecular changes having been induced by EBRT. However, normal tissue damage due to delivery of EBRT first could make the brachytherapy procedure difficult with increased risk of toxicity. It is, therefore, essential to know whether there are any significant differences in toxicities and treatment outcomes, especially acute urinary toxicity among the two treatment approaches.

This randomized feasibility study will look at the treatment arms according to the timing of HDR brachytherapy—either before or after EBRT—and their toxicity profiles. The study is called Timing of HDR brachytherapy with EBRT in Prostate CAncer (THEPCA). Assessment of acute and late toxicities and other parameters in these two arms at various time points will enable appropriate sequencing of EBRT and HDR therapy resulting in an optimal level of reduced toxicity. The treatments from both arms will be delivered with standard planning techniques. The incidence of grade 3 genitourinary toxicity is 1.6% in this cohort of patients [5]. Additionally, this feasibility study will also explore the challenges of image-guided radiotherapy planning between the two study arms. Provided a significant difference between the two treatment arms is achieved following final analysis, consideration will be made to use this to inform the development of a further pivotal study to look more deeply into the toxicity and other parameters related to the treatment.

### Trial Objectives

#### Primary Objective

The primary objective of this study is the prospective assessment of genitourinary toxicities related to the treatment sequence of HDR brachytherapy and EBRT.

#### Secondary Objectives

The secondary objectives of this study are to assess treatment outcomes, including biochemical response and survival, prospective assessment of gastrointestinal toxicities according to the treatment sequence of HDR brachytherapy and EBRT, and assessment of radiotherapy planning challenges, including image-guided radiotherapy.

#### Primary and Secondary Endpoints

The primary endpoint of this study is the presence of grade 3 genitourinary toxicity in patients. The secondary endpoints of this study are the presence of all other grades of genitourinary toxicity (ie, grades 1 and 2), the presence of gastrointestinal toxicity (ie, grades 1 to 4), prostate-specific antigen (PSA) recurrence-free survival, overall survival, and quality of life (QoL).

## Methods

### Trial Design

This study will be a randomized, two-arm trial in which intermediate and high-risk prostate cancer patients are treated with both HDR brachytherapy and EBRT. In Arm A, patients will receive HDR brachytherapy before EBRT. In Arm B (control arm), patients will receive EBRT before HDR brachytherapy. The assessment of the acute and late toxicities at various time points will be carried out. The treatment should start within 3 months from the randomization date. This trial has been registered with the International Standard Randomized Controlled Trial Number (ISRCTN) registry (ISRCTN: 15835424).

Toxicity will be assessed using the following tools:

1. International Prostate Symptom Score (IPSS)

2. International Index of Erectile Function Scale (IIEFS)

3. Functional Assessment of Cancer Therapy-Prostate (FACT-P), version 4

4. National Cancer Institute Common Terminology Criteria for Adverse Events (CTCAE), version 4, grading system

**Figure 1:**
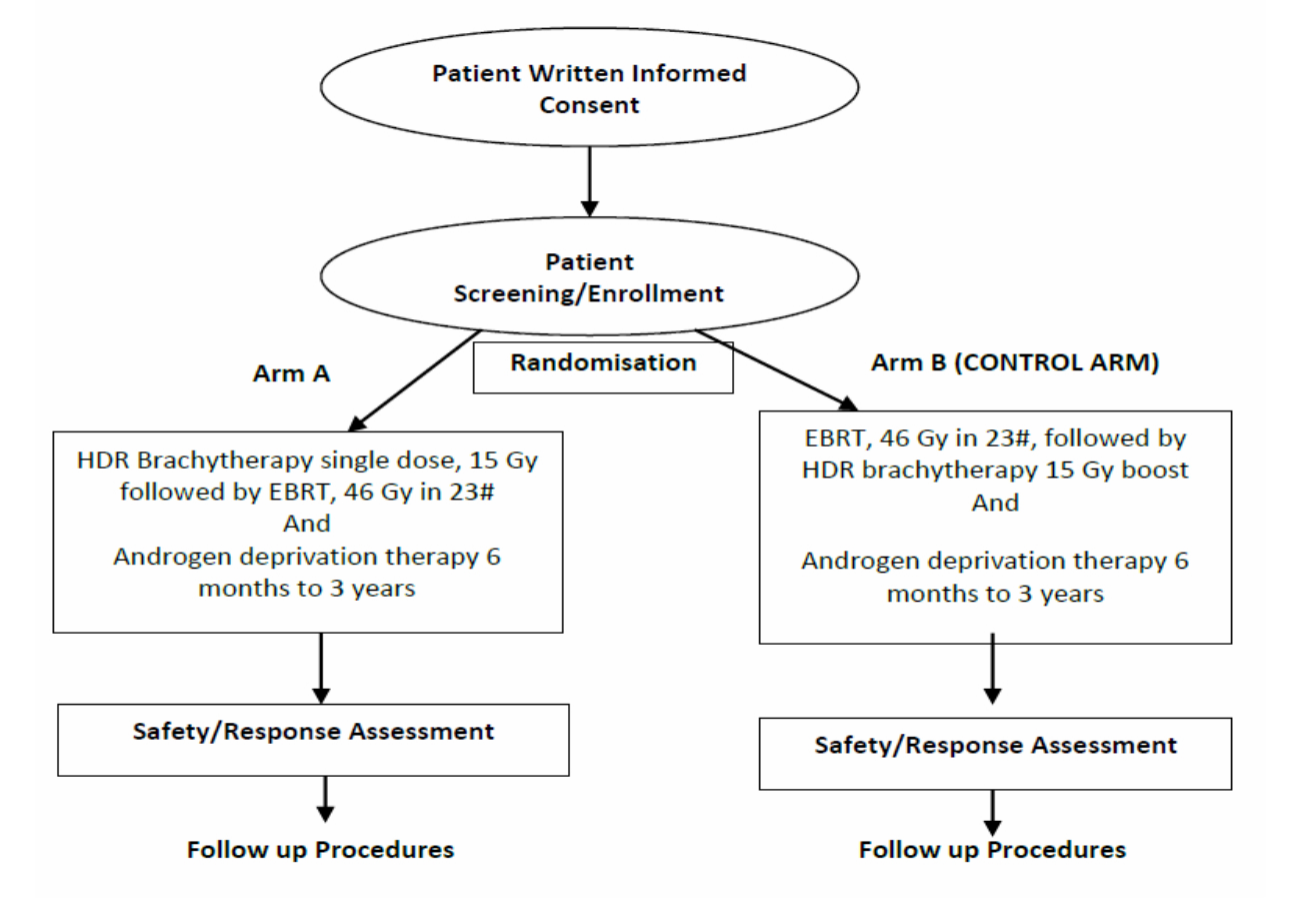
THEPCA study scheme design.

The THEPCA study scheme design is shown in Figure 1.

### Number of Participants and Participant Selection

A total of 50 patients will be recruited to the whole study, which includes both arms of the study for evaluation of the outcomes. It is estimated that the dropout rate due to screen failures will be approximately 10%. Inclusion and exclusion criteria for the study are shown in Textbox 1.

**Inclusion and exclusion criteria for the THEPCA study.**

Inclusion Criteria

- 18 years of age or older
- Histologically diagnosed prostate cancer (stages T1b-T3bN0M0)
- Any Gleason score
- Any PSA level
- Patient must be able to provide consent and fill in the questionnaires

Exclusion Criteria

- Previous transurethral resection of the prostate (TURP)/holmium laser enucleation of the prostate (HoLEP) laser prostatectomy
- Any metastatic disease
- An IPSS greater than 20
- Pubic arch interference
- Lithotomy position
- Anesthesia is not possible
- Rectal fistula
- Prior pelvic radiotherapy

### Study Procedures

#### Screening Procedures

Patients will undergo the following procedures as per the standard of care, the results of which will be communicated to the investigator for their review prior to approaching the potential participant: tumor staging (CT/MRI/bone scans), histological confirmation of diagnosis (Gleason score), and PSA measurement.

#### Informed Consent and Randomization Procedures

##### Overview

It is the responsibility of the investigator, or an appropriately trained person (ie, trained in Good Clinical Practice [GCP]) delegated by the investigator as documented in the site delegation log, to obtain written informed consent from each participant prior to any participation-/study-specific procedures. This will follow adequate explanation of the aims, methods, anticipated benefits, and potential hazards of the study.

The participant will be given ample time to consider giving their informed consent for the study—for this study, 24 hours will be given during which time the consenting physician will be reachable by phone to answer any questions. The date that the Participant Information Sheet (PIS) is given to the participant will be documented within the patient’s notes to ensure that sufficient time was given.

If, for some reason, the consenting physician is not accessible by phone and the participant wishes to speak with them, a second consent visit should be arranged.

The investigator, or other qualified person, will explain to the potential participant that they are free to refuse involvement with any part of the study, or alternatively may withdraw their consent at any point during the study for any reason.

If there is any further safety information which may result in significant changes in the risk/benefit analysis, the PIS and the Informed Consent Form (ICF) will be reviewed and updated accordingly. All participants that are actively enrolled in the study will be informed of the updated information and given revised copies of the PIS and ICF in order to confirm their wish to continue in the study.

##### Randomization

Randomization will be carried out by the clinical trials data manager within the Anglia Ruskin Clinical Trials Unit (ARCTU) at Anglia Ruskin University (ARU). ARCTU uses the Trans European Network ALEA (TENALEA) randomization service provided by the Trans European Network for Clinical Trial Services. This is an Internet-based randomization system, which will be set up for the study by ARCTU in accordance with the protocol. It will be a simple 1:1 ratio randomization, which will only be possible if a participant meets the inclusion criteria and not the exclusion criteria. The system stores the predetermined sequence of randomization—this list is not visible to the investigator or to the Anglia Ruskin Clinical Trials Unit. Once a patient has consented to take part in the trial, they will be randomly allocated to either Arm A or Arm B. The research nurse or investigator will log onto the Web browser application and enter the patient’s eligibility and stratification factors into the system. The study arm allocation is then returned to the investigator and to selected members of ARCTU and the study team. Please refer to the study scheme diagram in Figure 1.

#### Baseline Procedures

The following baseline procedures will be performed: physical examination, measurement of vital signs, and assessment of QoL baseline (IPSS, IIEFS, FACT-P, CTCAE questionnaires), hematology, biochemistry, concomitant treatment (eg, androgen deprivation therapy [ADT]), and PSA.

#### Treatment Modalities

#####  Brachytherapy Procedure

The brachytherapy procedure will be carried out at the surgical theaters in Southend University Hospital. The steps are as follows:

1. Patients will undergo prostate implantation under general or spinal anesthetic using a transrectal ultrasound-guided transperineal technique.

2. Imaging according to local practice using ultrasound, CT, and/or MRI will be undertaken.

3. The clinical target volume for prostate (CTVp) is defined by the prostate capsule and is extended to include any extra capsular or seminal vesicle disease. A volumetric expansion of 3 mm constrained to the rectum posteriorly is then added—this defines the planning target volume (PTV).

4. Catheter reconstruction and dwell time definition is then undertaken to provide a treatment plan for approval by the treating clinician.

5. Treatment is delivered once an optimized plan has been approved.

6. After completion of treatment in the brachytherapy room, the implant catheters and urinary catheter are removed—no anesthesia is required for this procedure.

7. The patient will return to the ward and may be discharged home later the same day or the following day.

##### Dose Prescription

A dose of 15 Gy will be given in a single treatment exposure defined at 100% isodose, which is the minimum tumor isodose to cover the PTV. PTV recommendations are as follows: the minimum dose received by 90% of PTV (D90) should be ≥15 Gy, and the volume of the target area receiving 100% of the prescribed dose (V100) should be ≥95%. See Table 1 for the risk-tolerance doses of the rectum and urethra.

**Table 1:** Organs at risk-tolerance doses.

Organ	Risk-tolerance dose
Rectum D2cc^a^	12 Gy
Rectum V100^b^	0 cc
Urethra D10^c^	<17.5 Gy
Urethra D30^d^	<16.5 Gy
Urethra V150^e^	0 cc

^a^Dose to 2 cm^3^(D2cc).

^b^Volume of target area receiving 100% of prescribed dose (V100).

^c^Dose covering 10% (D10) of the urethral volume.

^d^Dose covering 30% (D30) of the urethral volume.

^e^Volume of target area receiving 150% of prescribed dose (V150).

##### External Beam Radiotherapy

EBRT will be given to prostate and seminal vesicles only, using either intensity-modulated radiotherapy or volumetric-modulated arc radiotherapy (VMAT) to a dose of 46 Gy in 23 fractions over 4½ weeks. The dose-volume histogram (DVH) would be according to the local radiotherapy protocol. The gap between BT and EBRT, irrespective of their sequence, should not exceed 3 weeks. Therefore, the total radiotherapy treatment time should be up to 7½ weeks.

### Androgen Deprivation Therapy

Patients will receive neoadjuvant and adjuvant antiandrogen therapy from 6 months to 3 years according to the risk stratification of the disease as per the standard of care.

Table 2 shows the schedule of assessments throughout the study.

**Table 2:** Schedule of assessments during the study.

Steps and assessments	Pretreatment time points	Treatment time points		Posttreatment time points^a^				
	Screening^b^and consent	Baseline	Start of second treatment^c^	6 weeks	3 months	6 months	9 months	12 months
Informed consent	*							
Physical examination		*	*	*	*	*	*	*
Vital signs		*	*	*	*	*	*	*
QoL, IPSS, IIEFS, FACT-P, CTCAE^d^		*			*		*	*
Hematology		*						*
Biochemistry		*						*
Concomitant treatment (eg, ADT^d^)		*	*	*	*	*	*	*
Tumor staging	*							
Histological confirmation of diagnosis	*							
PSA^d^	*	*			*	*	*	*
RT^d^and brachytherapy dose				*				

^a^assessments performed after complete treatment

^b^screening procedures carried out as per standard of care

^c^start day of second treatment modality

^d^Quality of life (QoL), International Prostate Symptom Score (IPSS), International Index of Erectile Function Scale (IIEFS), Functional Assessment of Cancer Therapy-Prostate (FACT-P), Common Terminology Criteria for Adverse Events (CTCAE), androgen deprivation therapy (ADT), prostate-specific antigen (PSA), radiotherapy (RT).

### End of Study Definition

The definition of the end of the study is the point at which the last patient recruited has had the last visit at the end of the 1-year follow-up session.

### Participant Withdrawal

#### Overview

A patient may withdraw, or be withdrawn, from trial treatment for the following reasons:

1. If the patient has to undergo urinary catheterization for relieving blockage symptoms while undergoing EBRT and should not proceed further with HDR.

2. Any other unforeseen toxicity developed during RT treatment, and as a consequence the patient is unable to finish the protocol treatment.

The withdrawn patients will be followed as per protocol up to the end of year 1 from the time of completed treatment. With ongoing consent, patients should remain in the trial and be followed up according to the protocol visit schedule.

#### Withdrawal of Consent

Patients may withdraw their consent to participate in the trial at any time. If the patient explicitly states their wish not to contribute further data to the study, the investigator should inform the coordinating center in writing and the withdrawal of consent should be documented by the investigator in the patient’s case report form (CRF). However, data up to the time of consent withdrawal will be included in the data reported for the study.

Although the participant is not obliged to give the reason for withdrawing their consent, this information will help ascertain any trends related to trial procedures and may influence the protocol development in future projects.

### Laboratory Tests

All laboratory tests will be taken as per the standard of care within the local pathology department at Southend University Hospital National Health Service (NHS) Foundation Trust. Tests include full blood count (FBC), liver function tests (LFTs), urea, electrolytes, and PSA.

The samples will be collected by the trial nurse, labelled and logged in the CRFs, processed according to the local standard operating procedures (SOPs), and the results will be recorded in the CRFs.

### Pharmacovigilance

#### General Definitions

##### Adverse Event

An adverse event (AE) is any untoward medical occurrence in a participant to whom a medicinal product has been administered, including occurrences which are not necessarily caused by, or related to, that product. An AE can, therefore, be any unfavorable and unintended sign, including an abnormal laboratory finding, symptom, or disease temporarily associated with study activities.

##### Serious Adverse Event

A serious adverse event (SAE) fulfils at least one of the following criteria: (1) is fatal—results in death (NOTE: death is an outcome, not an event), (2) is life-threatening, (3) requires inpatient hospitalization or prolongation of existing hospitalization, (4) results in persistent or significant disability/incapacity, (5) is a congenital anomaly/birth defect, or (6) is otherwise considered medically significant by the investigator.

#### Investigator’s Assessment

##### Seriousness

The chief investigator (CI) or principal investigator (PI) responsible for the care of the participant, or in his absence an authorized medic within the research team, is responsible for assessing whether an event is serious according to the definitions given above.

##### Causality

The investigator must assess the causality of all serious adverse events in relation to the trial treatment according to the definitions given above.

##### Expectedness

The investigator must assess the expectedness of all SAEs according to the definitions given above. If the SAE is unexpected and related, then it needs immediate reporting.

##### Severity

The investigator must assess the severity of the event according to the following terms and assessments. The intensity of an event should not be confused with the term “serious” which is a regulatory definition based on participant/event outcome criteria.

1. Mild: intensity of an event is mild if some discomfort is noted, but without disruption of daily life.

2. Moderate: intensity of an event is moderate if discomfort is enough to affect/reduce normal activity.

3. Severe: intensity of an event is severe if it causes a complete inability to perform daily activities and lead a normal life.

####  Notification and Reporting of Adverse Events or Reactions

If the AE is not defined as *serious*, the AE is to be recorded in the study file and the participant is to be followed up by the research team. The AE is to be documented in the participant’s medical notes where appropriate, and in the CRF.

####  Notification and Reporting of Serious Adverse Events

Serious adverse events that are considered to be *related* and *unexpected* are to be reported to the sponsor within 24 hours of learning of the event and to the main research ethics committee (REC) within 15 days in line with the required time frame. For further guidance on this matter, please refer to Multimedia Appendix 1.

#### Urgent Safety Measures

The CI may take urgent safety measures to ensure the safety and protection of the clinical trial participants from any immediate hazard to their health and safety, in accordance with Regulation 30 of The Medicines for Human Use (Clinical Trials) Regulations 2004: SI 2004/1031. The measures should be taken immediately. In this instance, the approval from the licensing authority prior to implementing these safety measures is not required. However, it is the responsibility of the CI to inform the sponsor and main research ethics committee—via telephone—of this event immediately.

The CI has an obligation to inform the main ethics committee *in writing within 3 days*, in the form of a substantial amendment. The sponsor must be sent a copy of the correspondence with regard to this matter. For further guidance on this matter, please refer to Multimedia Appendix 1.

#### Annual Safety Reporting

The CI will send the Annual Safety Report (ASR) to the main REC using the National Research Ethics Service (NRES) template—the anniversary date is the date on the multicenter research ethics committee (MREC) “favorable opinion” letter—and to the sponsor.

####  Overview of the Safety Reporting Process/Pharmacovigilance Responsibilities

The CI has the overall pharmacovigilance oversight responsibility. The CI has a duty to ensure that pharmacovigilance monitoring and reporting is conducted in accordance with the sponsor’s requirements.

### Statistical Considerations

#### Primary Endpoint Analysis

Percentages will be compared using Fisher’s exact test. This analysis will be carried out after the end of the follow-up at 12 months.

#### Secondary Endpoint Efficacy Analysis

For the IPSS and IIEFS scale scores, the two means at each of the follow-up assessments will be compared using a two-sided permutation *t* test, and the 95% confidence limits for the difference between the means will be calculated using a bootstrap method. There will also be an assessment of trends in the scores over time using a repeated measures analysis of variance on the four follow-up scores, with the baseline score as a covariate. Prostate-specific antigen relapse-free survival will be estimated using the Kaplan-Meier method, with a test for the difference between the survival curves using the log-rank test. Cox proportional hazards multiple regression will also be used to assess the effects of covariates on survival. For this feasibility study, this analysis will be carried out after the end of the follow-up at 12 months, whereas for a main study a longer follow-up period would be considered.

#### Safety Endpoints

As the primary endpoint is concerned with adverse events, this will be a central concern of the primary endpoint analysis as described above—the analyses will be carried out after the end of the follow-up at 12 months.

#### Sample Size

In this feasibility study, the sample size has not been determined according to statistical principles, but is the number judged to be suitable for evaluating the suitability of the processes and procedures of running the study, and for assessing the patient experience and adherence in the study. To this end, two samples of 25 patients—50 overall—will be randomized to the two treatments.

#### Statistical Analysis

Although the sample size will be small, there will nevertheless be attempts to analyze the data in the same way as would be the case for a main study. However, this might not always be possible depending on the pattern of the outcomes and missing values. For descriptive statistical summaries, continuous data will be summarized using means, medians, standard deviations, interquartile ranges, and ranges. Categorical data will be summarized using counts and percentages. All statistical significance testing will be at the 5% significance level. For the IPSS and IIEFS scale scores, the two means at each of the follow-up assessments will be compared using a two-sided permutation *t* test using 1,000,000 random permutations, and the 95% confidence limits for the difference between the means will be calculated using a bootstrap method using 9999 resamplings. There will also be an assessment of trends in the scores over time using a repeated measures analysis of variance on the four follow-up scores with the baseline score as a covariate.

For categorical data based on adverse events, percentages will be compared using Fisher’s exact test. In this small study it will be possible to carry out the full combinatorial calculations for Fisher’s exact test, whereas in a main study, 10,000 random permutations will be obtained in a Monte Carlo approach. For differences between percentages, the 95% confidence limits will be obtained using Newcombe’s Hybrid Score Interval method. For the secondary analysis, prostate-specific antigen relapse-free survival will be estimated using the Kaplan-Meier method, with a test for the difference between the survival curves using the log-rank test—the *P* value will be obtained using a permutation test with 10,000 permutations. Cox proportional hazards multiple regression will also be used to assess the effects of covariates on survival, with model comparisons carried out using likelihood ratio tests. The analyses will be performed using the computer program R. All randomized participants will be included in the analyses. There are no planned interim analyses.

### Data Handling and Record Keeping

#### Confidentiality

The investigator has the responsibility to ensure that participant anonymity is protected and maintained. He/she must also ensure that participant identities are protected from any unauthorized parties. Information with regard to study participants will be kept confidential and managed in accordance with the Data Protection Act, NHS Caldicott Guardian, The Research Governance Framework for Health and Social Care and Research Ethics Committee Approval.

#### Study Documents

The list of study documents is shown in Textbox 2.

**Study documents required for the administration of the THEPCA study.**

- A signed protocol and any subsequent amendments

- Current/superseded Participant Information Sheets (as applicable)
- Current/superseded Informed Consent Forms (as applicable)
- Indemnity documentation from sponsor
- Conditions of sponsorship from sponsor
- Conditional/final research and development (R&D) approval
- Signed site agreement
- Ethics submissions/approvals/correspondence
- CVs of CI and site staff
- Laboratory accreditation letter, certification, and normal ranges for all laboratories to be utilized in the study
- Delegation log
- Staff training log
- Site signature log
- Participant identification log
- Screening log
- Enrolment log
- Monitoring visit log
- Protocol training log
- Correspondence relating to the trial
- Communication plan between the CI/PI and members of the study team
- SAE reporting plan for the study

#### Case Report Form

Project data collection will be managed by the Clinical Trials Unit data manager who will oversee recruitment and collection of data. The responsibility for data entry rests with the research nurse who is supported by the investigator. The ARCTU uses an online data management system called MACRO to design and manage electronic case report forms (eCRFs). The ARCTU will work together with the Southend study team to design and validate the data collection tools so that they are appropriate for this study. Once a patient is enrolled in the study, the research team can access these forms remotely through the Internet portal and study data will be entered and captured for the study.

All data will be in anonymized form—patients will be identifiable only by study number. Data will be remotely monitored by the ARCTU and discussed at data monitoring committee meetings. Any inconsistencies, validation errors, or inaccuracies will be reported to the lead investigator regularly. Once data collection is complete and the data has been validated, a data lock will be performed and analysis can begin.

#### Record Retention and Archiving

During the course of the research, all records are the responsibility of the chief investigator and must be kept in secure conditions. When the research trial is complete, it is a requirement of the Research Governance Framework and Trust Policy that the records be kept for a further 20 years.

#### Compliance

The CI will ensure that the trial is conducted in compliance with the principles of the Declaration of Helsinki (1996), and in accordance with all applicable regulatory requirements, including, but not limited to, the Research Governance Framework, Trust and Research Office policies and procedures and any subsequent amendments.

### Clinical Governance Issues

#### Ethical Considerations

This protocol and any subsequent amendments, along with any accompanying material provided to the participant in addition to any advertising material, will be submitted by the investigator to an independent research ethics committee. Written approval from the committee must be obtained and subsequently submitted to the Trust’s Research and Development Office to obtain final R&D approval.

#### Quality Control and Quality Assurance

##### Summary Monitoring Plan

The ARCTU will ensure that the project is carried out in accordance with the Research Governance Framework. All research team members will have GCP training before the research commences to ensure every aspect from trial design to dissemination is carried out in line with these principles. GCP is an international quality standard that is provided by the International Conference on Harmonisation (ICH), an international body that defines standards, which governments can transpose into regulations for clinical trials involving human subjects.

##### Audit and Inspection

The definition for *auditing* from section 1.6 of the ICH GCP Guideline is as follows: “A systematic and independent examination of trial related activities and documents to determine whether the evaluated trial related activities were conducted, and the data were recorded, analysed and accurately reported according to the protocol, sponsor's standard operating procedures (SOPs), Good Clinical Practice (GCP), and the applicable regulatory requirement(s).”

The THEPCA study may receive an audit by any of methods listed below:

1. A project may be identified via the risk assessment process.

2. An individual investigator or department may request an audit.

3. A project may be identified via an allegation of research misconduct or fraud, or a suspected breach of regulations.

4. Projects may be selected at random. The Department of Health states that Trusts should be auditing a minimum of 10% of all research projects.

5. Projects may be randomly selected for audit by an external organization. Internal audits will be conducted by a sponsor’s representative.

#### Noncompliance

Noncompliance, as described in the ICH GCP Guideline, can be defined as “a noted systematic lack of both the CI and the study staff adhering to SOPs/protocol/ICH-GCP, which leads to prolonged collection of deviations, breaches or suspected fraud.”

Noncompliance events may be captured from a variety of different sources including monitoring visits, CRFs, communications, and updates. The sponsor will maintain a log of the noncompliance events to ascertain if there are any trends developing which need to be escalated. The sponsor will assess the noncompliance events and implement a time frame of actions in which they need to be dealt with. Each action will be given a different time frame dependent on the severity. If the noncompliance events are not dealt with accordingly, the sponsor will agree on an appropriate action, including an on-site audit.

#### Trial Committees

##### Trial Management Group

A Trial Management Group (TMG) has been formed comprising the chief investigator, other lead investigators—clinical and nonclinical—and members of the data centers. The TMG will be responsible for the day-to-day running and management of the trial and will meet at least three times a year by teleconference.

##### Trial Steering Committee

The Trial Steering Committee (TSC) has membership from TMG plus independent members, including the chair. The role of the TSC is to provide overall supervision for the trial and provide advice through its independent chairman. The ultimate decision for the continuation of the trial lies with the TSC.

##### Independent Data Monitoring Committee

The Independent Data Monitoring Committee (IDMC) is the only group who sees the confidential, accumulating data from the trial. Reports to the IDMC will be produced by the Clinical Trials Unit (CTU) statisticians. The IDMC will meet within 6 months of the trial opening, with the frequency of meetings dictated by the IDMC.

##### Radiotherapy Quality Assurance Subgroup

The Radiotherapy Quality Assurance Subgroup developed the RT quality assurance (QA) plan and issued guidance on delivering RT in this trial.

## Results

Results from this feasibility trial will be available in mid-2016.

## Discussion

If the results from this feasibility trial show evidence that the sequence of treatment modality does affect the patients’ toxicity profiles, then funding would be sought to conduct a large, multicenter, randomized controlled trial.

## Multimedia Appendix 1

Information with regard to safety reporting.

## Abbreviations

ADT: androgen deprivation therapy
AE: adverse event
ARCTU: Anglia Ruskin Clinical Trials Unit
ARU: Anglia Ruskin University
ASR: Annual Safety Report
BT: brachytherapy
CI: chief investigator
CRF: case report form
CTCAE: Common Terminology Criteria for Adverse Events
CTU: Clinical Trials Unit
CTVp: clinical target volume for prostate
D2cc: dose to 2 cm3
D10: dose covering 10% of volume
D30: dose covering 30% of volume
D90: minimum dose received by 90% of planning target volume
EBRT: external beam radiotherapy
eCRF: electronic case report form
EORTC QLQ-C30: European Organisation for Research and Treatment of Cancer Quality of Life Questionnaire for Cancer Patients
EPIC: Expanded Prostate Cancer Index Composite
FACT-P: Functional Assessment of Cancer Therapy-Prostate
FBC: full blood count
GCP: Good Clinical Practice
HDR: high-dose rate
HDR-BT: high-dose rate brachytherapy
HoLEP: holmium laser enucleation of the prostate
ICF: Informed Consent Form
ICH: International Conference on Harmonisation
IDMC: Independent Data Monitoring Committee
IGRT: image-guided radiotherapy
IIEFS: International Index of Erectile Function Scale
IMRT: intensity-modulated radiotherapy
IPSS: International Prostate Symptom Score
LDR: low-dose rate
LFT: liver function test
MREC: multicenter research ethics committee
NHS: National Health Service
PI: principal investigator
PIS: Participant Information Sheet
PSA: prostate-specific antigen
PTV: planning target volume
QA: quality assurance
QLQ PR25: Quality of Life Questionnaire for Prostate Cancer Patients
QoL: quality of life
R&D: research and development
RCT: randomized controlled trial
REC: research ethics committee
RT: radiotherapy
SAE: serious adverse event
SOP: standard operating procedure
TENALEA: Trans European Network ALEA
TMG: Trial Management Group
TSC: Trial Steering Committee
TURP: transurethral resection of the prostate
V100: volume of the target area receiving 100% of the prescribed dose
V150: volume of the target area receiving 150% of the prescribed dose
VMAT: volumetric-modulated arc radiotherapy
