# Supplementary material for: Timing of High-Dose Rate Brachytherapy With External Beam Radiotherapy in Intermediate and High-Risk Localized Prostate CAncer (THEPCA) Patients and Its Effects on Toxicity and Quality of Life: Protocol of a Randomized Feasibility Trial
Source: JMIR Res Protoc. 2015 Apr 29;4(2):e49. doi: 10.2196/resprot.4462 (PMC4430680; doi:10.2196/resprot.4462)
Supplement: Supplementary file 1 [file resprot_v4i2e49_app1.pdf]

Appendix 1 – Information with regards to Safety Reporting

	Who	When	How	To Whom
**SAE**	Chief Investigator	-Report to Sponsor within 24 hours of learning of the event<br><br>-Report to the MREC within 15 days of learning of the event	SAE Report form for Non-CTIMPs, available from NRES website.	Sponsor and MREC
**Urgent Safety Measures**	Chief Investigator	Contact the Sponsor and MREC Immediately<br><br>Within 3 days	By phone<br><br>Substantial amendment form giving notice in writing setting out the reasons for the urgent safety measures and the plan for future action.	Main REC and Sponsor<br><br>Main REC with a copy also sent to the sponsor. The MREC will acknowledge this within 30 days of receipt.
**Progress Reports**	Chief Investigator	Annually (starting 12 months after the date of favourable opinion)	Annual Progress Report Form (non-CTIMPs) available from the NRES website	Main REC
**Declaration of the conclusion or early termination of the study**	Chief Investigator	Within 90 days (conclusion)<br>Within 15 days (early termination)<br><br>*The end of study as defined in the protocol*	End of Study Declaration form available from the NRES website	Main REC with a copy to be sent to the sponsor
**Summary of final Report**	Chief Investigator	Within one year of conclusion of the Research	No Standard Format However, the following Information should be included:-<br>Where the study has met its objectives, the main findings and arrangements for publication or dissemination including feedback to participants	Main REC with a copy to be sent to the sponsor
